# Endoscopic Treatment of Postoperative External Biliary Fistula in a Patient Operated on for Hepatic Injury Due to Multiple Trauma

**DOI:** 10.1155/DTE.3.67

**Published:** 1996

**Authors:** Marco de Monti, Davide Sonnino, Marina Gorziglia, Giorgio Redaelll, Marcello Scarpis

**Affiliations:** 1 Department of General Surgery General Hospital of Menaggio Como Italy; 2 Digestive Endoscopy Service General Hospital of Menaggio Como Italy; 3 S.S.U.Em. 118 General Hospital of Menaggio Como Italy; 4 Via Derna 5 Milano I-20132 Italy

## Abstract

After surgery for hepatic injury as a result of blunt abdominal trauma from a motorcycle accident, an external biliary fistula developed in a young patient. The authors describe the rapid and complete healing of the fistula by use of a nasobiliary catheter. These findings emphasize the importance of endoscopic operative technique for postoperative and traumatic external biliary fistulas.


*Diagnostic and Therapeutic Endoscopy*, Vol. 3, pp. 67–72
Reprints available directly from the publisher
Photocopying permitted by license only

© 1996 OPA (Overseas Publishers Association)
Amsterdam B.V. Published in The Netherlands
by Harwood Academic Publishers GmbH
Printed in Singapore

### Endoscopic Treatment of Postoperative External Biliary Fistula in a Patient Operated on for Hepatic Injury Due to Multiple Trauma

MARCO DE MONTI*,†, DAVIDE SONNINO, MARINA GORZIGLIA, GIORGIO REDAELLL and MARCELLO SCARPIS

*Department of General Surgery (D.S., M.G., G.R.), Digestive Endoscopy Service (M.S.), and S.S.U.Em. 118 (M.D.M.), General Hospital of Menaggio, Como, Italy*

*(Received 26 January 1996; In final form 20 March 1996)*

**After surgery for hepatic injury as a result of blunt abdominal trauma from a motorcycle accident, an external biliary fistula developed in a young patient. The authors describe the rapid and complete healing of the fistula by use of a nasobiliary catheter. These findings emphasize the importance of endoscopic operative technique for postoperative and traumatic external biliary fistulas.**

*Keywords:* External biliary fistula, endoscopic retrograde cholangiopancreatography, nasobiliary catheter

### INTRODUCTION

External biliary fistula is a possible complication of hepatic trauma or hepatic surgery. It can also be a result of elective biliary surgery for lithiasis, neoplasm, or echinoccosis [1,2]. Its incidence is estimated to be 0.1 to 0.3% of all biliary operations [3]. Biliocutaneous fistulas due to spontaneous drainage of biliary or hepatic abscesses are extremely rare [4]. Untreated blunt hepatic trauma can cause external fistulas in about 4% of all patients [5].

Causes of postoperative biliocutaneous fistulas include residual lithiasis, distal stenosis, premature removal of Kehr tubes, and iatrogenic injury or accidental ligation of the common biliary duct [6]. Fistulous drainage may cause significant fluid and electrolyte losses, requiring prolonged hospitalization.

Surgical therapy of external biliary fistulas is often associated with high morbidity (50%) [7] and mortality due to procedures performed in inflamed and septic tissues.

Endoscopic operative procedures represent a valid and often successful treatment for these kinds of complications in biliary surgery: endoscopic retrograde cholangiopancreatography (ERCP) permits extremely precise diagnosis and allows biliary drainage through

*Corresponding author.

†Present address: Via Derna 5, I-20132 Milano, Italy.

a nasobiliary tube or endoprosthesis. Successful endoscopic therapy has been reported after endoscopic sphincterotomy [8,9] or drainage by use of nasobiliary catheters [10] or after positioning plastic or metal stents [11–13].

The authors report a case of external biliary fistula subsequent to an operation for hepatic injury in a patient with blunt abdominal trauma. Endoscopic positioning of a nasobiliary drainage tube permitted complete healing of the fistula within a few days.

### CASE REPORT

A 17-year-old male patient was involved in a motorcycle accident and was admitted to the emergency department exhibiting a right humerus fracture and abdominal blunt trauma. His arterial blood pressure could not be obtained; an abdominal ultrasound scan demonstrated a massive hemoperitoneum. As a result, laparatomy was immediately performed. The discovery of multiple dilacerations of the VIth, VIIth, and VIIIth liver segments suggested the extension of the laparotomy to a thoracolaparotomy. Intraperitoneal blood losses were evaluated as 4.5 liters; however, 2.2 liters were reinfused by an automatic blood intraoperative transfusion machine. Multiple hemostatic sutures were placed to avoid extensive hepatectomy. The patient was subsequently admitted to the critical care unit. On the 3rd postoperative day, biliary leakage from the abdominal drainage tube became evident. The leakage averaged approximately 800 ml in a 24-hr period. On the 16th postoperative day, surgical osteosynthesis of the humerus was performed. At the same time, an ERCP was performed, which demonstrated contrast leakage on the VIIIth segment intrahepatic biliary duct. Biliary tracts appeared extremely narrow due both to the young age of the patient and the fact that they were empty. Cholelithiasis was not found (Fig. 1). A papillosphincterotomy was performed, and a nasobiliary tube was placed directly in the injured biliary duct. One day later, only 40 ml of biliary leakage was registered, and 600 ml had been drained from the nasobiliary tube. Afterward no more bile leaked from the abdominal drainage tubes. A trans-nasobilary tube cholangiography, performed 5 days later, demonstrated the persistence of leakage of contrast material (Fig. 2). Another cholangiography, performed 12 days after ERCP, demonstrated complete healing of the fistula (Fig. 3).

After 40 days of hospital stay, the patient was completely recovered and was discharged.

### DISCUSSION

The possibility of spontaneous resolution of biliocutaneous fistulas directly depends on the drainage capability of the main biliary tract. Fistulas will be sustained until the obstacle is removed. Persistence of biliary leakage around biliary ducts generally causes definitive stenosis. Prompt choledochal drainage prevents biliary leakage complications, which may occur early on or at a later stage [14]. Decompression of the biliary tract causes a significant reduction of fistula output (up to 90 to 95%) [5], facilitating internal drainage and fistula closure in a shorter time. It is reported that untreated bile leakage spontaneously resolves in an average of 33 days (ranging from 3 to 110 days), with a high incidence of complications such as infections, sepsis, and respiratory failure [15]. Healing of nasobiliary drained fistulas is reported to occur between 2 and 3 weeks without any complications [2,5]. First, the procedure requires a correct diagnosis using ERCP to identify the precise location of the fistula. The next step involves the wedging of the nasobiliary catheter as distally as possible in the biliary tract, which is directly responsible for nourishing the fistula. The nasobiliary catheter must be maintained in continuous aspiration. Additionally, in our patient, we preferred to perform a papillosphincterotomy to decrease the pressure in the biliary tree and also to allow the bile to drain from the left side of the biliary tree, which had not been drained by the catheter.

In 1979, Agrawal *et al.* [16] described the first endoscopic diagnosis of biliary fistulas. In 1983, O'Raihilly *et al.* [17] successfully cured a fistula by endoscopic sphincterotomy. The first endoscopic treatment of post-traumatic biliocutaneous fistula was

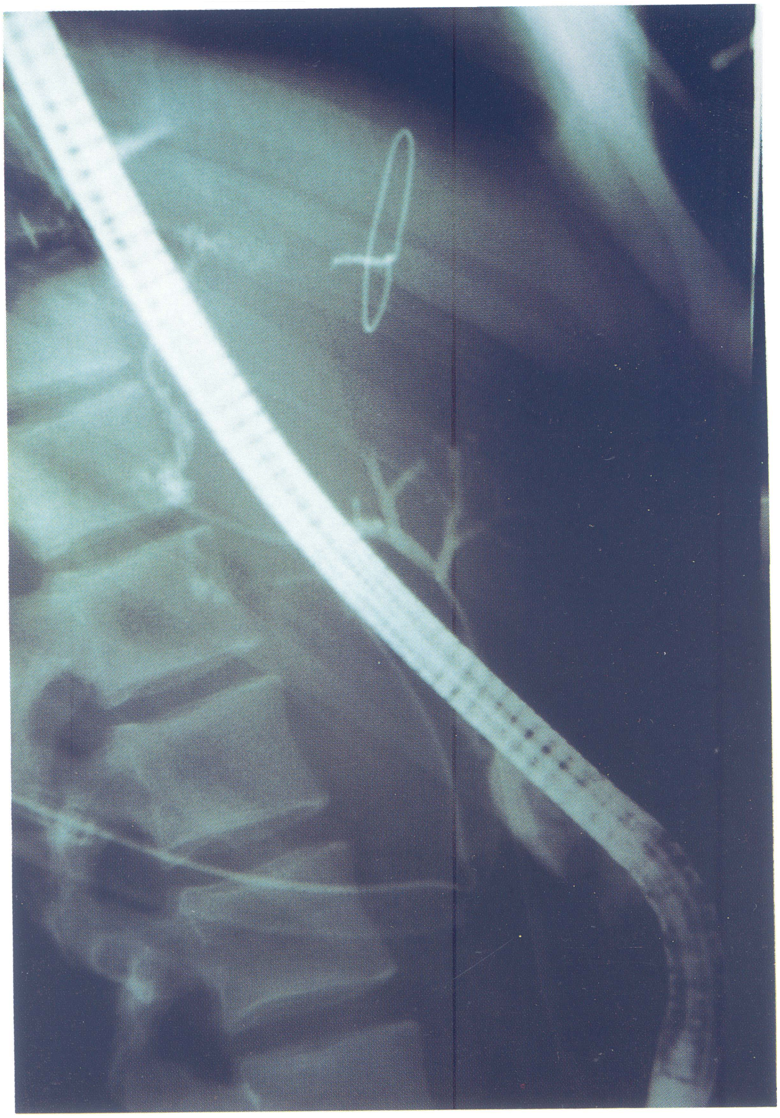

FIGURE 1 ERCP: contrast material leakage in the biliary branch of the VIIIth segment.

performed by Burmeister *et al.* [18] in 1985, with a nasobiliary drainage tube. The first use of endoprostheses in external biliary fistulas was described in 1986 by Huibregtse *et al.* [19] and by Smith *et al.* [11] and, subsequently by Devier *et al.* [20] in 1987.

ERCP permits a complete study of the biliary tree, except for fistulas caused by complete ligature of choledochus: however, patients with these fistulas are candidates for surgery. In all other patients biliocutaneous fistulas, an 86% success rate is reported [3,6].

Some authors [3] suggest inserting an endoprosthesis instead of the nasobiliary tube because of physiologic duodenal bile drainage. Other authors [14] report a 100% success rate with use of sphincterotomy associated with a nasobiliary tube.

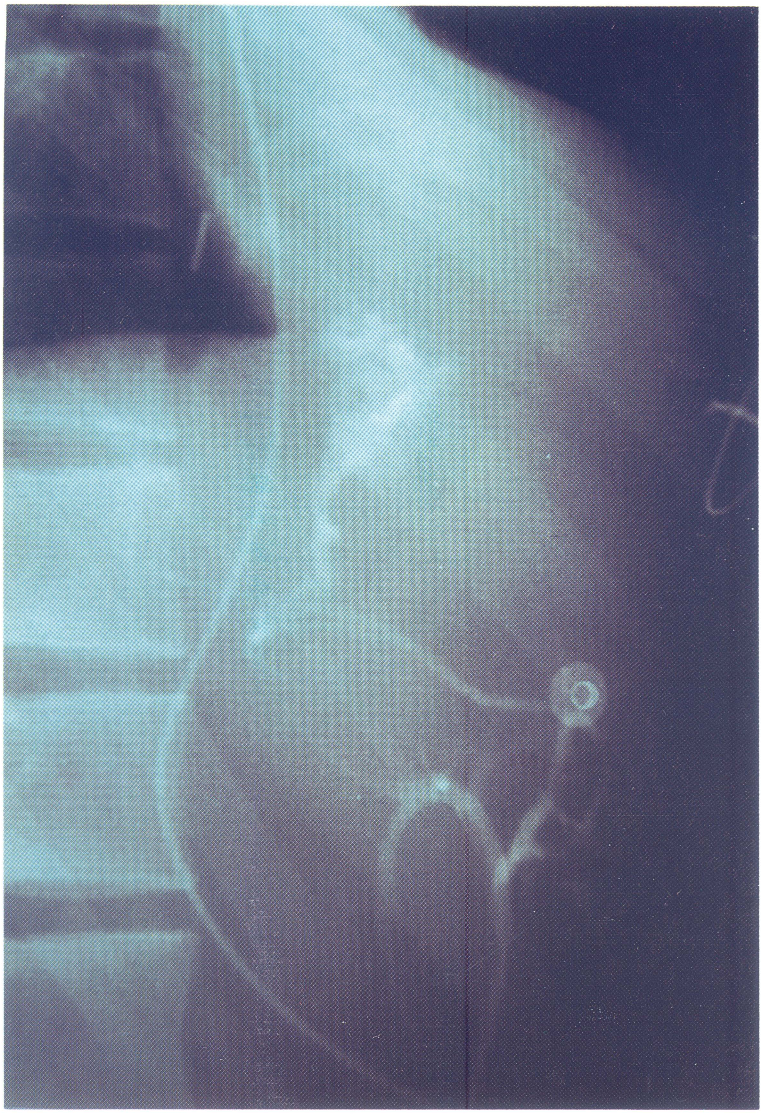

FIGURE 2 First trans-nasobiliary tube cholangiography (5th post-ERCP day): the biliary fistula of the VIIIth segment is still evident.

### CONCLUSIONS

Operative endoscopy and nasobiliary drainage provide excellent results and is supported by a breadth of international literature, which suggests that endoscopic operative techniques must be the treatment of choice for postoperative and traumatic external biliary fistulas. Moreover, endoprostheses can be used when nasobiliary tube aspiration is not functioning, especially in fistulas caused by neoplastic involvement of the biliary tract.

### *References*

[1] Magistrelli, P., Masetti, R., Coppola, R., *et al.* (1989). Value of ERCP in the diagnosis and management of pre and postoperative biliary complications in hydatid disease of the liver, *Gastrointest Radiol*, **14**, 315–320.

[2] Iscan, M., Duren, M. (1991). Endoscopic sphincterotomy in

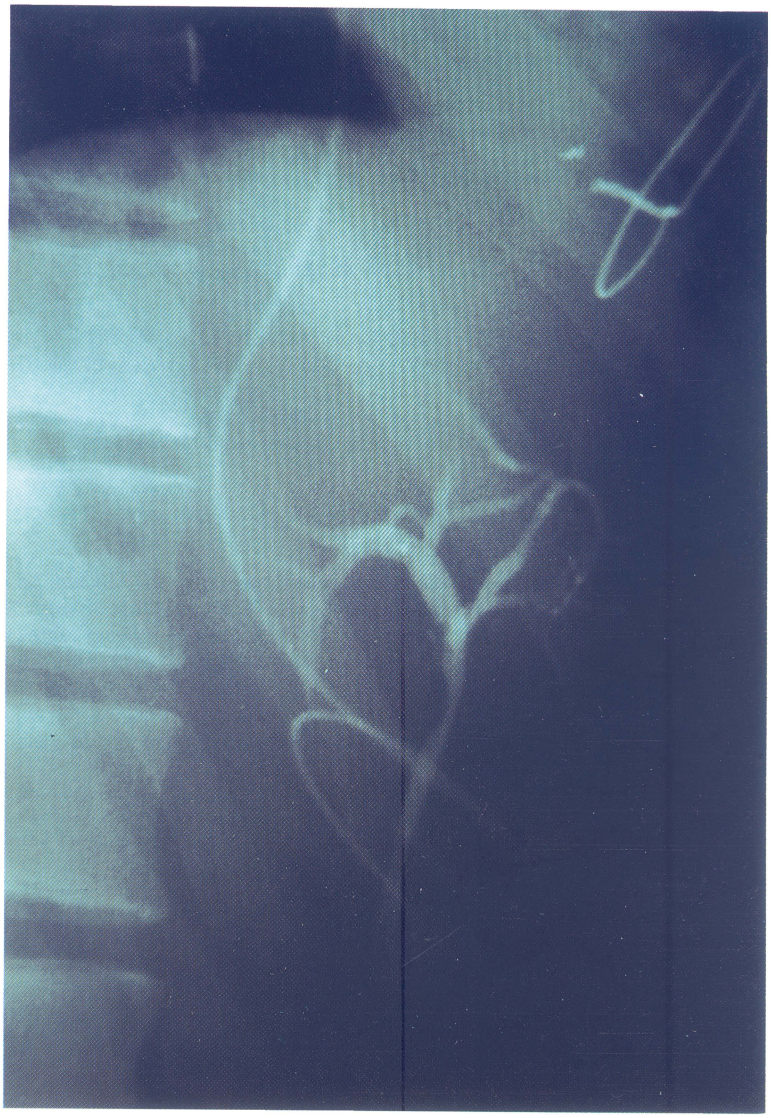

FIGURE 3 Second trans-nasobiliary tube cholangiography (12th post-ERCP day): the contrast material leakage is not evident anymore.

the management of postoperative complications of hepatic hydatid disease, *Endoscopy,* **23**, 282–283.

[3] Coppola, R., Riccioni, M. E., Ciletti, S., *et al.* (1992). Diagnosi e trattamento endoscopico delle fistole biliari post-operatorie, *Giorn Ital End Dig,* **15**, 25–36.

[4] Leung, J. W. C., Sung, Y. J., Li, M. K. W., *et al.* (1988). Biliary stenting as treatment for a spontaneous bile leak, *Gastrointest Endosc,* **83**, 1431–1432.

[5] Scioscia, P. J., Dillon, P. W., Cilley, R. E., *et al.* (1994). Endoscopic sphincterotomy in the management of post-traumatic biliary fistula, *J Pediatr Surg,* **29**, 3–6.

[6] Huibregste, K. (1989). Endoscopic biliary and pancreatic drainage, *Stattgart: George Thieme,* **4**, 2.

[7] Hadjis, N. S., Blumgart, L. H. (1988). Injury to segmental bile ducts, *Arch Surg,* **123**, 351–353.

[8] Ponchon, T., Galles, J. F., Valette, P. J., *et al.* (1989). Endoscopic treatment of biliary tract fistulas, *Gastrointest Endosc,* **35**, 490–498.

[9] Del Olmo, L., Merono, E., Moreira, V. F., *et al.* (1988). Successfull treatment of postoperative external biliary fistulas by endoscopic sphincterotomy, *Gastrointest Endosc,* **34**, 307–309.

[10] Burmeister, W., Koppen, M. O., Wurbs, D. (1985). Treatment of a biliocutaneous fistula by endoscopic insertion of a nasobiliary tube, *Gastrointest Endosc,* **31**, 279–281.

[11] Smith, A. C., Schapiro, R. H., Kelsey, P. B., *et al.* (1986). Successful treatment of nonhealing biliary cutaneous fistulas with nasobiliary stents, *Gastroenterology,* **90**, 764–769.

[12] Goldin, E., Katz, E., Wengrower, D., *et al.* (1990). Treatment of fistulas of the biliary tract by endoscopic insertion of endoprostheses, *Surg Gynecol Obstet,* **170**, 418–423.

[13] Davids, P. H. P., Groen, A. K., Rauws, E. A. J., *et al.* (1992). Randomized trial of self-expanding metal stents versus polyethylene stents for distal malignant biliary obstruction, *Lancet,* **340**, 1488–1492.
[14] Biagini, M., Balestri, B., Dei, A., *et al.* (1994). Fistole biliari iatrogene: ruolo dell'endoscopia, *Giorn Ital End Dig,* **17**, 43–46.
[15] Hollands, M. J., Little, J. M. (1991). Post-traumatic bile fistulae, *J Trauma,* **31**, 117–120.
[16] Agrawal, R. M., Mitre, R., Brodmerkel, G. J. Jr, *et al.* (1979). The diagnosis of post-cholecystectomy cystic duct fistula utilizing ERCP, *Endoscopy,* **3**, 193–199.
[17] O'Rahilly, S., Duignan, J., Lennon, J. R., *et al.* (1983). Successful treatment of a postoperative external biliary fistula by endoscopic papillotomy, *Endoscopy,* **15**, 68–69.
[18] Burmeister, N., Roppen, M. O., Wurbs, D. (1985). Treatment of a biliocutaneous fistula by endoscopic insertion of a nasobiliary tube, *Gastrointest Endosc,* **31**, 279–281.
[19] Huibregste, K., Katon, R., Tygat, G. N. (1986). Endoscopic treatment of postoperative biliary stricture, *Endoscopy,* **18**, 133–137.
[20] Devier, J., Van Gansbeke, D., Ansay, J., *et al.* (1987). Endoscopic management of a post-traumatic biliary fistula, *Endoscopy,* **19**, 136–139.

